# Characteristics and determinants of loss to follow-up among tuberculosis (TB) patients who smoke in an industrial state of Malaysia: a registry-based study of the years 2013-2017

**DOI:** 10.1186/s12889-022-13020-3

**Published:** 2022-04-01

**Authors:** Zatil Zahidah Sharani, Nurhuda Ismail, Siti Munira Yasin, Yuslina Zakaria, Asmah Razali, Nur Atiqah Rochin Demong, Mariam Mohammad, Zaliha Ismail

**Affiliations:** 1 Department of Public Health Medicine, Faculty of Medicine, Universiti Teknologi MARA (UiTM) Sungai Buloh Campus, 47000 Sungai Buloh, Selangor Malaysia; 2 Biomedical Epidemiology Unit, Special Resource Centre, Institute for Medical Research (IMR), National institute of Health (NIH) Setia Alam, 40170 Shah Alam, Selangor Malaysia; 3 Department of Pharmacology, Faculty of Pharmacy, Universiti Teknologi MARA (UiTM) Puncak Alam Campus, 42300 Sungai Buloh, Selangor Malaysia; 4 Sector TB/Leprosy, Disease Control Division, Ministry of Health, 62590 Putrajaya, Malaysia; 5 Department of Technology and Supply Chain Management Studies, Faculty of Business and Management, Universiti Teknologi MARA, 42300 Puncak Alam, Selangor Malaysia

**Keywords:** Loss to follow-up, Tuberculosis, Tobacco smoking, Treatment outcome

## Abstract

**Background:**

The increased risk of loss to follow-up among TB smokers raises concern over the secondary spread within the community. This study aimed to determine the factors associated with loss to follow-up among TB patients who smoke.

**Methods:**

All registered TB patients who smoke in the state of Selangor between 2013 and 2017 via the Malaysian Tuberculosis Information System (MyTB) database were included for analysis. TB patients who smoke were considered those who are “current smoker” during the notification, while loss to follow-up was defined as a TB patient who had interrupted treatment for 2 months or longer. There were 3 main variable domains included for analysis: sociodemographic profiles, disease profiles, and comorbidities. Logistic regression analysis was used to identify determinants of loss to follow-up among TB patients who smoke.

**Results:**

A total of 14.1% (*N* = 813) of TB patients who smoke loss to follow-up. The determinants of loss to follow-up among TB smokers were working age population aged 32-41 and 42-53 years old (AOR 1.08; 95%CI 1.23,2.08) and (AOR 1.44; 95%CI 1.11,1.87) respectively, Malaysian nationality (AOR 2.34; 95%CI 1.66,3.30), patients staying in urban area (AOR 1.55; 95% CI 1.23,1.97), income level less than RM2160 (AOR 1.59; 95% CI 1.14,2.20), un-employed (AOR 1.30; 95%CI 1.09-1.55), have low education level i.e., secondary school education, primary school education and no formal education (AOR 1.60; 95%CI 1.22,2.10), (AOR 1.73; 95%CI 1.16,2.57) and (AOR 2.29; 95% CI 1.57,3.33) respectively, previously treated TB cases (AOR 2.19; 95% CI 1.71,2.81), active TB case detection methods (AOR 2.06; 95%CI 1.40,3.02), moderate lesion x-ray (AOR 1.60; 95%CI 1.13,2.27) and HIV positive (AOR 1.36; 95%CI 1.02,1.82). All the significant factors gave rise to the final model of determinants, with a predictability of 67.2% (95% CI 65.0,69.3).

**Conclusions:**

The high proportion of loss to follow-up among TB patients who smoke highlight the importance of providing early risk detection that examines the three main domains of risk factors such as socioeconomic, disease profiles and comorbidities. Potential integrated intervention should aim to reduce the proportion of smoking among TB patients through the stop smoking programme together with directly observed therapy (DOT).

## Background

### TB current situation

An estimated of 10 million cases of tuberculosis (TB) occurred in 2018, leading to 1.3 million deaths worldwide [1]. In Malaysia, TB is the leading cause of death (mortality rates fluctuate from 4.8-6.2 cases per 100,000 population) from a single infectious disease; TB ranked above HIV/AIDS, dengue fever and malaria in the 5-year period from 2012 to 2016 [2]. With the current trend of the TB notification rate, it is projected that the incidence of TB will continue to increase through 2030 [3]. There are 11 states in the Peninsular of Malaysia and Selangor recorded the highest number of TB cases. Selangor is an industrialised state with > 90% urban population, creating more than 802,000 employment opportunities as reported in 2019 [4].

TB is a mandatory national notifiable infectious disease under the Prevention and Control of Infectious Disease Act 1988. All suspected and confirmed cases of TB should be reported and notified to the nearest district health office by submission of the notification form. In our country, data from the notification form are entered into the national TB registry by the health inspectors of the respective district health officer. The Malaysian Tuberculosis Information System (MyTB) is a web-based application administered by the Ministry of Health Malaysia to record activities related to the notification, registration, investigation, and treatment of TB diseases in all states in Malaysia.

Smoking and TB remain major public health challenges globally. Tobacco smoking is responsible for 20% of the global burden of tuberculosis and will be responsible for a total of 18 million new cases and 40 million deaths in the 2010-2050 period [5]. It is projected that TB incidence will increase up to 7% if we incorporate the effect of smoking compared to the effect without smoking [5]. Numerous studies have identified smoking as a risk factor for the development of TB [6] and has a significant association with undesirable treatment adherence and loss to follow-up [7]. Smoking has also been associated with more extensive lung disease and delayed sputum conversion even after 2 months of treatment in both current smokers and ex-smokers, which makes their treatment outcome worse [8]. A higher proportion of smokers has been noted among TB patients compared to the general population in countries such as Indonesia, Africa, and India [9–11]. In Malaysia, the prevalence of current smokers among TB patients was 34.0% in 2012 [12] compared to only 22.7% among the general population [13].

### Loss to follow-up from TB treatment

In addition to smoking, loss to follow-up from TB treatment is also another threat faced by the TB control team in Malaysia. Patients with incomplete treatment for TB will become a significant economic burden on the government, with an average of RM901.63 (215.49USD) per patient four times higher compared to the cost of completed treatment [14]. The extra cost is normally attributed to hospital stays and patient care for complicated TB cases [14]. The prevalence of loss to follow-up varies among different countries and ranges from 2.5 to 44.9% [15]. A very high prevalence of 44.9% has been observed in rural northern Mozambique, where loss to follow-up rates are a very serious problem [16]. While in Malaysia, the prevalence of loss to follow-up ranged from 4.0 to 4.8% in the years 2010-2015 among the general TB population [2]; and this number has increased to 5.6% according to the latest study [17]. Studies from many other countries have shown a significant association between smoking and tendency to loss to follow-up; however, there are limited data on the baseline proportion of loss to follow-up among those who smoke in the TB population. A local study in Penang found that smoking among TB patients is significantly associated with poorer treatment outcomes and had increased risk for loss to follow-up by OR 7.17 compared to non-smokers [18]. In Hong Kong, studies have concluded that smoking is the key contributor to loss to follow-up, with a doubled risk compared to that of TB patients who never smoked [19]. Similar findings have also been found in studies from Morocco and Tehran, where smokers have a double and triple increased likelihood for loss to follow-up in their TB treatment, respectively [20, 21].

### Determinants of loss to follow-up

Previous studies have identified several reasons and risk factors for loss to follow-up among TB patients, including smoking, alcohol use, comorbidities (HIV and diabetes mellitus), accessibility to a healthcare centre, socioeconomic factors (age, sex, education level, and income), and poor family support [22–24]. Loss to follow-up is also common among those who previously defaulted on TB treatment and among relapse case, which occur mostly during the intensive phase of treatment [25]. The evidence for a connection between smoking and loss to follow-up, however, is inconclusive. Some studies have hypothesized that smokers with TB disease are less likely to comply with their TB treatment [26], while other studies have found that smokers have low levels of concern for their health. The behaviour of delaying seeking medical care at a more severe phase of illness and non-compliance among smokers may result in their worse prognosis [27]; however, there is no solid conclusion for this hypothesis.

Despite of the knowledge on the impact of smoking and incomplete treatment among TB patients, there have been limited studies that examine these two issues. Intervention should be emphasized towards reducing the prevalence of tobacco use among TB patients due to their negative impact on TB treatment outcome including loss to follow-up. An early identification of smoking TB patients who are at higher risk for loss to follow-up is a way to ensure these population receive specific intervention that aid them to quit smoking and improve their adherence on TB treatment. This present study aimed to determine the factors associated with loss to follow-up among TB patients who smoke in a 5-years cohort (2013-2017) of patients registered in the Selangor MyTB database.

## Methods

### Study setting, inclusion, and exclusion criteria

This was a cross-sectional study that utilized data from the National MyTB database version 2.1 in 2013 to 2017 from the Disease Control Division, Ministry of Health. TB data from all states in Malaysia are consolidated at the national level for the surveillance database; however, in this study, we included only TB patients who smoke who were registered in the Selangor MyTB database from 2013 until 2017. The state of Selangor was selected based on its high TB incidence i.e., 5071 cases in 2018 [28]. Both Malaysians and non-Malaysians were included in this study. The exclusion criteria in this study includes cases initially registered as TB but ultimately diagnosed as something other than TB disease, cases with missing data on treatment outcomes and duplicated cases. The other TB treatment outcome such as died, treatment failure and outcome not evaluated were also removed to avoid biases due to the overlapping possibilities of the treatment outcome. For example, those who died from TB and those who failed TB treatment could have been those who loss to follow-up and vice versa. We also excluded cases with multidrug-resistant TB (MDR-TB), as the treatment outcome definition for MDR-TB cases is different from the non-MDR-TB classification. TB treatment outcome and disease classification were defined according to the World Health Organization definition and reporting framework [29].

### Operational definition

According to the Clinical Practice Guideline (CPG) for the management of tuberculosis by Ministry of Health Malaysia and the definition and reporting framework of the WHO [29, 30], treatment outcomes of TB can be categorized into successful treatment outcomes (cured and completed treatment) and unsuccessful treatment outcomes (loss to follow-up, treatment failure, death). In this study, the outcomes were divided into “loss to follow-up” and “successful treatment outcome”. A loss to follow-up (case) was defined as TB patients who interrupted treatment for ≥2 months or longer before the end of the treatment period [29]. For the comparison group, successful treatment outcome includes TB patient who have been cured (defined as a negative sputum culture in the last month of treatment and on at least one previous occasion) and treatment completion (defined as completing treatment without meeting the criteria for cured or treatment failure).

While, the other TB treatment outcomes that were not included in this study were treatment failure (TB patients whose sputum smear or culture was positive in the fifth month or later during treatment), died (TB patients who died before starting a treatment or during treatment was classified as a death, irrespective of the cause) and outcome not evaluated (TB patients with no assigned outcome or transferred out cases to another country whose the outcomes were not known).

Smoking status was obtained by confirming whether the patient was a “current smoker”. Current smoker was defined as person who currently smokes at least one tobacco product every day (daily smoker) or less than daily (occasional smoker) [31]. Further information on the frequency of smoking habit, number of cigarettes smoked per day, or the type of tobacco product used were not available in the database.

### Sample size

Sample size was estimated using Epi-Info software based on an alpha of 0.05, a power of 80%, and a design effect of 1. By taking the highest proportion of loss to follow-up among TB smokers at 11.6% by Leung et al., 2015 [19], the minimum sample size required is 158 cases. However, in this study, all registered TB patients who smoke in the Selangor MyTB 2.1 database from 2013 to 2017 were considered for the analysis, making up the total sample size of 4795.

### Variable

Sixteen variables related to the objective of the study were examined. The three domains of the independent variables were sociodemographic profiles (age, sex, nationality, ethnicity, locality, education level, personal median monthly income, and occupation), disease profiles (TB anatomical category, TB case category, method of TB case detection, BCG scar, chest radiography status and sputum status) and comorbidity (diabetes mellitus and HIV). The cut off point for the income level at RM2160 (515.48 USD) was based on the median monthly income of Malaysian population in year 2017 [32].The outcomes variable in this study were divided into “loss to follow-up” and “successful treatment outcome (cured and completed treatment)”.

### Data management

**Fig. 1:**
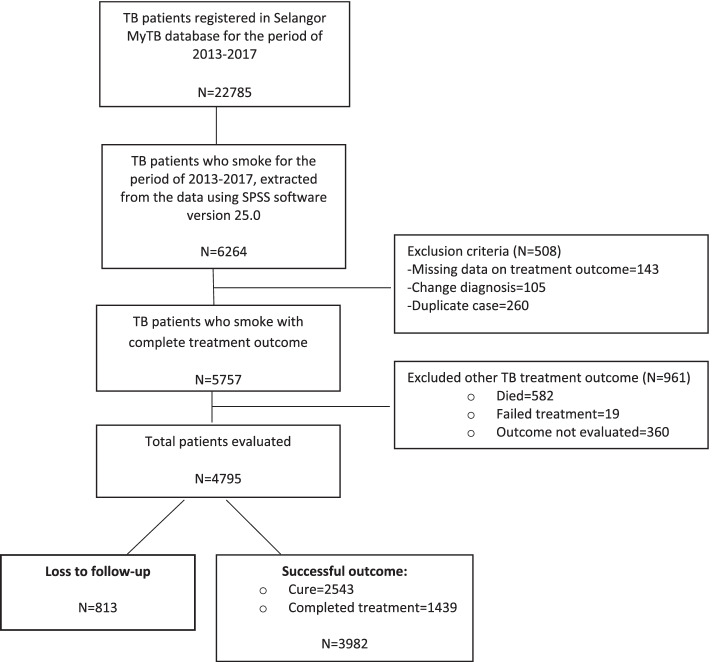
Flow diagram of data management flow

Data extraction flow is summarized in a flow diagram (Fig. 1). Data cleaning and processing were performed using R programming in view of the large amount of data. Redundant data were reviewed, and missing data were treated with data imputation. A total of 1469  cases who meet the exclusion criteria were excluded from the database. Data were kept in two backup storage in both hard and soft copies.

### Confidentiality

Patient identification and information in the database remained anonymous and were kept confidential. Data will be stored for 5 years in a password-protected hard disk and will be destroyed after that.

### Statistical analysis

Data were analysed using the SPSS statistical software package, version 25.0. The descriptive analysis of the three main independent variable domains is presented in the form of a frequency table. Simple logistic regression and multiple logistic regressions (MlogR) were performed to estimate the risk of loss to follow-up among TB patients who smoke. Significant results with *p* < 0.05 from the univariable analysis were considered in MlogR. Multivariable analysis was performed using backward LR. A value of p < 0.05 from the final logistic model was considered statistically significant. Adjusted odds ratios (AORs) were used to present the results. The presence of multicollinearity and interaction between the independent variables was checked.

## Result

A total of 22,785 TB patients were registered in the Selangor MyTB database from 2013 to 2017. Out of these 22,785 TB patients, 27.5% (*N* = 6264) were smokers and 10.3% (*N* = 2346) had loss to follow-up. After excluding cases with missing data on treatment outcome (*N* = 143), patients who changed diagnosis other than TB (*N* = 105) and duplicated cases (*N* = 260), the proportion of TB patients who smoke with complete treatment outcome is 5757. By using the number of total TB patients who smoke with complete treatment outcome (*N* = 5757), 69.2% of TB patients who smoke had successful treatment outcome in which 44.2% were cured and 25% completed TB treatment. While, for the unsuccessful treatment outcome, 14.1% loss to follow-up, 10.1% died, 0.3% failed treatment and 6.3% have outcome not evaluated. A total of 4795 patients were included for analysis, since patients with treatment outcome of “died”, “failed treatment” and “outcome not evaluated” were removed from the comparison group (Fig. 1).

### Characteristics of TB patients who smoke

The mean age of the TB patients who smoke was 41.41 ± 14.34 years and ranged from 8 to 88 years old. The majority were male 95.7% (*N* = 4588), Malaysian citizens 89.4% (*N* = 4287), lived in the urban area 83.0% (*N* = 3982), and of Malay ethnicity 57.5% (*N* = 2759). Most of the patients had an education level equal to at least to secondary school 64.7% (*N* = 64.7%), employed 63.4% (*N* = 3040) and reported monthly income level of less than RM 2160 87.3% (*N* = 4186).

In terms of the disease profile, 91.9% (*N* = 4409) were new TB cases, and 88.8% (*N* = 4260) were pulmonary TB cases. A quarter of the TB patients who smoke had DM comorbidity 20.3% (*N* = 971), while 6.9% (*N* = 330) were HIV positive. Majority of TB patients who smoke were monitored under the directly observed therapy (DOT) during the intensive phase 4478 (93.4%), however the number reduced to 80% (*N* = 3835) during the continuation phase (Table 1).

**Table 1:** Characteristics of the TB patients who smoke and are registered in Selangor (*N* = 4795)

Variables		***N*** = 4795	%
Sociodemographic profile			
Age	< 32	1386	28.9
	32-41	1184	24.7
	42-53	1204	25.1
	> 53	1021	21.3
Sex	Male	4588	95.7
	Female	207	4.3
Nationality	Malaysian	4287	89.4
	Non-Malaysian	508	10.6
Locality	Urban	3982	83.0
	Rural	813	17.0
Ethnicity	Malay	2759	57.5
	Chinese	745	15.5
	Indian	601	12.5
	Others	182	3.8
	Non-Malaysian	508	10.6
Education level	Higher education	787	16.4
	Secondary school	3103	64.7
	Primary school	398	8.3
	No education	507	10.6
Income level	≤RM2160	4186	87.3
	>RM2160	609	12.7
Employment status	Employed	3040	63.4
	Un-employed	1755	36.6
Disease profile			
TB anatomical location	Pulmonary TB	4260	88.8
	Extra-Pulmonary TB	535	11.2
TB case category	New case	4409	91.9
	Recurrent case	386	8.1
TB case detection	Active	168	3.5
	Passive	4627	96.5
BCG scar	Yes	4193	67.4
	No	602	12.6
X-ray status (*N* = 4728)	No Lesion	412	8.6
	Minimal lesion	2824	58.9
	Moderate lesion	1387	28.9
	Severe lesion	105	2.2
Sputum status (*N* = 4718)	Negative	1449	30.2
	Positive	3269	68.2
Comorbidity			
HIV status (*N* = 4405)	Yes	330	6.9
	No	4075	85.0
DM status	Yes	971	20.3
	No	3824	79.7
DOT for TB treatment			
DOT during the intensive phase	Yes	4478	93.4
	No	317	6.6
DOT during the continuation phase	Yes	3835	80.0
	No	960	20.0
Treatment outcome (all outcome, *N* = 5757)			
Cured		2543	44.2
Completed TB treatment		1439	25.0
Died		582	10.1
Loss to follow-up		813	14.1
Failed treatment		19	0.3
Outcome not evaluated		360	6.3

### Determinants of loss to follow-up

Variables were analysed using simple logistic regression and multiple logistic regression, as illustrated in Table 2 and Table 3. After considering the effect of confounding and interactions, the variables that significantly contributed to loss to follow-up among TB patients who smoke were the working age group, aged 32-41 and 42-53 years old (AOR1.08; 95%CI 1.23,2.08) and (AOR1.44; 95%CI 1.11,1.87) respectively, Malaysian nationality (AOR 2.34; 95%CI 1.66,3.30), patients staying in urban area (AOR 1.55; 95% CI 1.23,1.97), monthly income level of less than RM2160 (515.82 USD) (AOR 1.59; 95% CI 1.14,2.20), un-employed (AOR 1.30; 95%CI 1.09-1.55), have lower education level i.e., secondary school education, primary school education and no formal education (AOR 1.60; 95%CI 1.22,2.10), (AOR 1.73; 95%CI 1.16,2.57) and (AOR 2.29; 95% CI 1.57,3.33) respectively, previously treated TB cases (AOR 2.19; 95% CI 1.71,2.81), active TB case detection methods (AOR 2.06;95%CI 1.40,3.02), having moderate lesion on the chest x-ray (AOR 1.60; 95%CI 1.13,2.27) and patient who tested positive for HIV (AOR 1.36; 95%CI 1.02,1.82)(Table 3). All the significant factors gave rise to the final model of determinants, with a predictability of 67.2% (95% CI 65.0-69.3).

**Table 2:** Univariable analysis of loss to follow-up among TB patients who smoke and are registered in Selangor

Variables		Crude OR	95% CI		*P*-Value*
			Lower	Upper	
Sociodemographic profile					
Age	< 32	1.32	1.04	1.67	0.025
	32-41	1.82	1.44	2.29	< 0.001
	42-53	1.42	1.15	1.82	0.002
	> 53	1			
Sex	Male	1.21	0.81	1.80	0.335
	Female	1			
Nationality	Malaysian	1.66	1.24	2.20	< 0.001
	Non-Malaysian	1			
Locality	Urban	1.25	1.01	1.54	0.042
	Rural	1			
Ethnicity	Malay	1			
	Chinese	0.48	0.37	0.63	< 0.001
	Indian	1.34	1.08	1.65	0.007
	Others	1.14	0.78	1.65	0.503
	Non-Malaysian	0.57	0.43	0.76	< 0.001
Education Level	No formal education	1.92	1.41	2.28	< 0.001
	Primary school	1.51	1.07	2.15	0.020
	Secondary school	1.79	1.41	2.28	< 0.001
	Higher education	1			
Income level	≤2160	2.27	1.70	3.04	< 0.001
	> 2160	1			
Employment status	Employed	1.38	1.18	1.61	< 0.001
	Un-employed	1			
Disease profile					
TB anatomical location	Extrapulmonary	1			
	Pulmonary	1.13	0.88	1.44	0.346
TB case categories	New case	1			
	Previously treated cases	2.73	2.18	3.43	< 0.001
TB case detection	Passive	1			
	Active	2.27	1.63	3.19	< 0.001
BCG status	Yes	1			
	No	0.68	0.53	0.87	0.003
X-ray status					
	No lesions	1			
	Minimal lesions	1.06	0.79	1.42	0.685
	Moderate lesions	1.44	1.06	1.94	0.019
	Severe lesions	1.27	0.72	2.24	0.406
Sputum status	Negative				
	Positive	1.28	1.08	1.52	0.004
Comorbidity					
HIV status	No	1			
	Yes	1.78	1.38	2.31	< 0.001
DM status	No	1			
	Yes	0.82	0.68	0.99	0.048

Test used: Simple Logistic Regression Analysis

*Significant at 0.05

**Table 3:** Determinants of loss to follow-up among TB patients who smoke and are registered in Selangor

Variable		Crude OR	95% CI	*P*-value	AOR	95% CI	*p*-value^a^
Age	< 32	1.32	1.04-1.67	0.025	1.08	0.83-1.42	0.537
	32-41	1.82	1.44-2.29	< 0.001	1.60	1.23-2.08	0.001
	42-53	1.42	1.15-1.82	0.002	1.44	1.11-1.87	0.007
	> 53	1			1		
Nationality	Malaysian	1.66	1.24-2.20	< 0.001	2.34	1.66-3.30	0.001
	Non-Malaysian	1			1		
Locality	Urban	1.28	1.04-1.57	0.023	1.55	1.23-1.97	< 0.001
	Rural	1					
Income level	≤RM2160	2.21	1.65-2.95	< 0.001	1.59	1.14-2.20	0.006
	>RM2160	1					
Employment status	Employed	1			1		
	Un-employed	1.38	1.18-1.61	< 0.001	1.30	1.09-1.55	0.004
Education Level	High Education	1			1		
	Secondary school	1.73	1.34-2.49	< 0.001	1.60	1.22-2.10	0.001
	Primary school	1.3	0.92-1.84	0.136	1.73	1.16-2.57	0.007
	No education	1.82	1.34-2.49	< 0.001	2.29	1.57-3.33	< 0.001
TB case categories	New case	1					
	Previously treated cases	2.58	2.07-3.22	< 0.001	2.19	1.71-2.81	< 0.001
TB case detection	Passive	1					
	Active (screening)	2.286	1.64-3.18	< 0.001	2.06	1.40-3.02	< 0.001
X-ray status	No lesions	1			1		
	Minimal lesions	1.06	0.79-1.42	0.685	1.17	0.84-1.63	0.363
	Moderate lesions	1.44	1.06-1.94	0.019	1.60	1.13-2.27	0.008
	Severe lesions	1.27	0.72-2.24	0.722	1.17	0.61-2.26	0.641
HIV status	Yes	1.78	1.38-2.31	< 0.001	1.36	1.02-1.82	0.037
	No	1					

*AOR* Adjusted odds ratio, *CI* Confidence interval, *B* Regression coefficient, Nagelkerke *R*^2^ = 0.086, Hosmer and Lemeshow test = 0.603, Classification = 83.0% correct, AUC = 0.672 (95%CI: 65.0-69.3)

^a^Test used: Multiple Logistic Regression Analysis (Method Backward LR; B Constant = −4.276, Model assumption are met Interaction considered in the model, No Multicollinearity)

## Discussion

In this study, we highlighted the high prevalence of smoking among TB patients in Selangor during the period 2013-2017. Almost one-third of the TB patients in this study cohort were current smokers, 27.5% (95% CI: 25.2,28.8), compared to only 22% among the adult general population in Malaysia in 2015 [13]. Previous studies conducted in Malaysia [18, 33] and other countries, such as Indonesia, Africa, and India [9–11], have reported similar findings, with a higher prevalence of smokers being found among TB patients compared to the general population. This finding is in line with the projection that described the impact of tobacco smoking on the increasing number of TB cases worldwide [5]. This current study also shows that the proportion of loss to follow-up among TB patients who smoke is higher than the general TB patients registered in Selangor, with outcomes of 14.1 and 10.3%, respectively. Smoking behaviour among TB patients by itself has a poor prognosis for TB treatment outcomes. TB patients who smoke and loss to follow-up from TB treatment will experience worse outcomes. Therefore, it is important to examine the factors associated with loss to follow-up among TB patients who smoke to optimize their treatment adherence and assist them in quitting smoking.

### Factors associated with loss to follow-up

Adherence to TB treatment influences the outcome of patients and has important role on the prevention of multidrug resistance (MDR-TB) [34]. Other study has shown that majority of TB patients who loss to follow-up had sputum samples that were smear positive when they returned for retreatment care, which indicates a high risk of transmission to the others [35]. Despite of patient supervision in the form of the directly observed therapy (DOT) that is currently being delivered in our healthcare setting, the rate of loss to follow-up remained unimproved.

Working age group, aged 32 to 53 years old were found to have higher risk for loss to follow-up compared to the older age group > 53 years old, which is different from the general TB population where older age have higher rate of loss to follow-up [36, 37]. Other sociodemographic factors that contributed to the loss to follow-up among TB patients who smoke were those residing in an urban area, having a low education level and a low-income level [median income for working population < RM2160 (515.48 USD)] and un-employed. Similar finding was found in a qualitative study performed in urban Morocco, where a low income and a low level of education were barrier resources among TB patients which led to loss to follow-up. The reasons were due to lack of money for transportation, the need to work despite illness, and no one aiding to obtain medication [35]. This shows that socioeconomic support plays important roles in ensuring the continuation of TB treatment especially among the working age population. Despite the full subsidization of anti-TB treatment to all TB patients, some out-of-pocket expenditures still exist, especially cost related to transportation. An average of RM439.42 (104.87 USD) out-of-pocket money per patient has been estimated in order to complete a 6-month TB treatment [38]. Any intervention that could reduce the cost of TB treatment will help to improve patients’ compliance with TB treatment.

Other studies from Hong Kong and Morocco have found that male sex and being a non-religious person have significant associations with smoking habits and loss to follow-up from TB treatment [7, 26]. This could account for the large observed differences in the proportion of males and females in this study; however, sex was not included in the final model in this study. The influence of religious belief on the effect of patient adherence to TB treatment could not be quantified, as religious status was not available in the database.

Under the domain disease profiles, patients with a history of previously being treated for TB had an almost double risk of loss to follow-up compared to new TB cases. It has been reported that previous experience with TB is a risk factor for loss to follow-up only when there was a previous history of defaulting treatment [7]. This could be related to the smoking habits, as studies conducted among TB patients who smoke in Hong Kong have revealed a significant association between retreatment cases among current smokers and TB patients who never smoke [19]. The complex psychosocial factors of smoking may explain its association with loss to follow-up and non-adherence; however, in this study, we did not address the underlying mechanism. Additional studies from other countries, such as Sudan, Morocco, and Brazil, also found a significant association between retreatment cases and TB loss to follow-up with ORs ranging from 3.2 to 6.5; this indicates that patients who had been defaulting their treatment will be at higher risk of loss to follow-up from their TB treatment again [35, 39, 40].

This study also found that TB patients who smoke and who were detected through active screening methods had a double-risk AOR of 2.047 (95% CI 1.206-3.473) for loss to follow-up compared to those who were detected through passive detection methods. This finding could be the result of the implementation of the national guideline for systematic screening for TB high-risk groups, such as TB/HIV comorbidities, inmate prisoners, diabetes patients, elderly individuals, and patients in methadone replacement therapy since 2015, where the majority of these high-risk groups were significantly associated with unfavourable TB treatment outcomes [2]. This action was intended to improve TB detection rates and to provide early TB treatment to them. Most detected TB comorbidities, for example, TB/HIV and TB/DM, are known to be predictors of poor TB treatment outcomes in many studies. Studies from Brazil, Kenya and Peru have found that HIV-infected patients are at higher risk of loss to follow-up than are HIV-negative patients [25, 41, 42]. The outcomes are similar for diabetes mellitus (DM); where clinical evidence has found DM to be a significant risk factor for poor TB treatment outcomes, including treatment default. The literature suggested that DM is significantly associated with the development of adverse drug reactions and delayed sputum conversion at the end of 2 months of treatment [43], which explains the high loss to follow-up rates among TB/DM patients in certain countries, including Kuwait and Brazil [44]. In this study, TB patients who smoked and had HIV were the significant predictors for loss to follow-up, while DM comorbidity were not included in the final model.

The lower association of loss to follow-up among TB patients who smoke with DM may be due to the increased TB detection rate and earlier TB treatment initiation among DM patients under the National Diabetes Programme [2]. Patients are frequently followed up for their chronic DM management which concurrently improved their overall adherence on TB treatment [12]. There is also collaborative activity between the National TB Control Programme and the National HIV/AIDS Control Programme to improve the surveillance, TB-HIV management, and treatment outcome of TB-HIV patients, however more effort is needed to improve patients’ compliance to TB treatment.

In Malaysia, national TB treatment guideline strongly recommends using patient-centred case management and utilizes the DOT (Directly observed therapy) strategy when treating people with TB. All newly diagnosed TB patients are compulsory to take their TB treatment under direct supervision of DOT during the intensive phase. The DOT supervisors could be healthcare workers, family members, NGOs, and community volunteers. Statistics have found that more patients experience lost to follow-up when supervised by NGOs and volunteers than when supervised by family members and healthcare workers [2]. While literature from other studies showed that patients who do not adhere to their treatment and loss to follow-up are largely unsupervised or supervised outside the chest clinic. In this study, the percentage of patients on DOT decreased from 93.4% during the intensive phase to 80.0% in the continuation phase. The reduction of percentage is worrying, as this could be the proportion of TB patients who loss to follow-up. Many patients who do not receive DOT will usually stop taking their medications after 2 months because they feel better or are less symptomatic [45]. Therefore, it is crucial to ensure that the selection of DOT supervisors is appropriate depending on the patients’ risk factors. Other common significant factors associated with non-adherence to DOT are poor knowledge towards TB and its treatment, the cost of transportation for DOT at every visit and the distance of the DOT centre from individual’s home [46].

Addressing smoking issues among TB patients requires a strategic plan on its own. A study performed among TB patients who smoke in Penang showed a poor score of tobacco use knowledge and its health consequences in general among newly diagnosed patients [33]. Most patients report that they are not informed about the impact of continued smoking on TB outcomes and have only received general health information and not TB-specific information [9]. Evidence on the effects of smoking cessation on TB treatment outcomes, especially on loss to follow-up is limited. It is also not well known whether quitting smoking during TB treatment would have an immediate impact and produce similar outcomes as those of individuals who have never smoked. However, a study performed by Wang and Shen in Hong Kong found that TB patients without smoking cessation are twice as likely to loss to follow-up from TB treatment than are those who achieve cessation (OR 2.03; 95% CI 0.99,4.18). This outcome is similar to a local study performed in Malaysia, which found that TB patients who receive tobacco cessation intervention during DOT have a lower rate of loss to follow-up than TB patients in the usual care group [47]. Therefore, initiating tobacco cessation intervention during DOT will improve patient’s adherence to TB treatment and benefit their overall treatment outcome.

### Strength and limitations

The findings from this study provide knowledge about the characteristics and determinants of loss to follow-up among TB patients who smoke. It also highlights the high proportion of loss to follow-up among TB patients who smoke for the attention of stakeholders to implement integrated intervention by addressing both TB and smoking issues. Other items of importance are the findings on the influence of social determinants among smokers on TB treatment defaults, such as education and economic status, and those on TB burden, especially in urban areas. The large sample size taken from the registered TB patients in the Selangor MyTB database from 2013 to 2017 allowed the generalizability of the study findings to the general TB population in Malaysia. However, this study has several limitations. Due to the massive data, advanced data software such as R software or STATA is required for data management and processing. The completeness of the database due to a high proportion of missing data affects the analysis for certain variables (HIV status, sputum status and chest X-ray status with missing values ranging from 1.3-8.1%). There were also important missing variables from the database that could not be analysed such marital status, alcohol use and drug abuse status, which limits our factor association analysis. Due to the limited information in the database in terms of smoking characteristics, we were unable to see the temporality of the associations between different smoking statuses among the patients (current smoker, ex-smoker, quit smoker) throughout TB treatment. Some TB patients might stop smoking after being diagnosed with TB; therefore, their smoking status should no longer be current smoker. Our study also lacks details on the timely duration of patient treatment to quantify at which phases of treatment should we intensify our supervision to prevent loss to follow-up.

### Recommendations

The reduction of the smoking prevalence among TB patients could be achieved through strengthening the inter-sectoral collaboration between units in the TB healthcare management system and reinforcing the communication or educational counselling quality between patients and health care providers. Integrated TB-tobacco cessation intervention programme must be initiated in TB chest clinics to cater to both issues holistically and should be included in the National Strategic Plan of TB for its better implementation.

Further observational studies with primary data collection are highly recommended. Another area for further research is to develop a prognostic scoring tool for an earlier detection of the high-risk group to loss to follow-up among those who smoke in the TB population based on the determinants that have been identified from this study. Extra supervision is required for TB patients who smoke and have high score for loss to follow-up.

## Conclusion

This study identified determinants for loss to follow-up among TB patients who smoke and focused on patients’ socioeconomic profiles, disease profiles and comorbidities. The logistic model showed that the risk of loss to follow-up could be predicted for those aged 32-53 years old, Malaysian, reside in an urban area, have no or low levels of education, have a low income, are a retreatment case, are a TB case detected through active methods, had moderate lesions on chest radiograph and has HIV comorbidity. Early risk detection for combined smoking cessation intervention and supervision through DOT should be provided to reduce smoking prevalence among TB patients and ultimately improve TB treatment outcomes, specifically regarding loss to follow-up.

## Data Availability

The dataset used and analysed in this current study is available from the corresponding author on reasonable request with permission from the Disease Control Division, Ministry of Health.

## Abbreviations

TB: tuberculosis
MyTB: Malaysian Tuberculosis Information System
DOT: directly observed therapy
DM: diabetes mellitus
HIV: human immune deficiency virus
AOR: adjusted odds ratio
COR: crude odds ratio
SD: standard deviation
